# Parasite proteostasis and artemisinin resistance

**DOI:** 10.21203/rs.3.rs-2926003/v1

**Published:** 2023-05-15

**Authors:** Melissa Rosenthal, Caroline Ng

**Affiliations:** University of Nebraska Medical Center; University of Nebraska Medical Center

## Abstract

The continued emergence and spread of resistance to artemisinins, the cornerstone of first line antimalarials, threatens significant gains made toward malaria elimination. Mutations in Kelch13 have been proposed to mediate artemisinin resistance by either reducing artemisinin activation via reduced parasite hemoglobin digestion or by enhancing the parasite stress response. Here, we explored the involvement of the parasite unfolded protein response (UPR) and ubiquitin proteasome system (UPS), vital to maintaining parasite proteostasis, in the context of artemisinin resistance. Our data show that perturbing parasite proteostasis kills parasites, early parasite UPR signaling dictate DHA survival outcomes, and DHA susceptibility correlates with impairment of proteasome-mediated protein degradation. These data provide compelling evidence toward targeting the UPR and UPS to overcome existing artemisinin resistance.

## Introduction

Malaria was responsible for a reported 247 million cases and 619,000 deaths in 2021, of which over 90% were caused by *Plasmodium falciparum*^1^. Implementation of artemisinin-based combination therapies (ACTs), the current first-line treatment for malaria, have contributed significantly to the approximately 11.7 million malaria deaths averted from 2000–2021^1^. Upon activation by heme released during parasite hemoglobin digestion^2–4^, artemisinin and derivatives (collectively referred to as artemisinins) non-specifically alkylate adjacent proteins, causing widespread protein damage to the parasite^5–8^. Alarmingly, resistance to artemisinins has been detected in Asia, South America, and Africa^1,9–27^. Clinically, artemisinin resistance is defined as parasite clearance half-life greater than 5 h following treatment with artemisinin monotherapy or an ACT^10^. In vitro, artemisinin resistance is defined as greater than 1% survival in the ring-stage survival assay (RSA)^28^. Resistance in Southeast Asian parasites is strongly associated with point mutations in Kelch13 (PF3D7_1343700)^10,29^, and C580Y is the most prevalent resistance-associated mutation in the Greater Mekong Subregion of Southeast Asia^30,31^.

The exact role of Kelch13 in artemisinin resistance is an ongoing area of study. Two non-mutually exclusive hypotheses have been put forward to explain Kelch13-mediated artemisinin resistance: (1) decreased artemisinin activation via reduced hemoglobin digestion, and (2) enhanced stress response to counter artemisinin-mediated protein and lipid damage. Knock sideways studies point to a role for Kelch13 in endocytosis of hemoglobin, as parasites in which > 60%^32^ of Kelch13 is mislocalized display both reduced uptake of fluorescent dextran^33^ and lower abundance of hemoglobin-derived peptides^34^. Omics studies point to a role for Kelch13 in the parasite stress response. Transcriptomics of artemisinin resistant clinical isolates were found to upregulate genes involved in protein folding, protein repair, and proteasome subunits^35^. In addition, the lab-adapted clinical isolate Cam3.II Kelch13 R539T and its isogenic counterparts Cam3.II Kelch13 WT and Cam3.II Kelch13 C580Y were examined by transcriptomics and proteomics, revealing that artemisinin-resistant (Kelch13 mutant) parasites express higher levels of genes involved in the ubiquitin proteasome system (UPS), redox, and intracellular vesicles^36^. Collectively, these transcriptomics and proteomics data point to a role for the unfolded protein response (UPR) in Kelch13-mediated artemisinin resistance.

We propose that parasite protein homeostasis, also referred to as proteostasis, is vital for parasite survival to DHA and other peroxides and impacts both Kelch13- and non-Kelch13-mediated artemisinin resistance mechanisms. Proteostasis is a reflection of the equilibrium between the total amount of proteins to be degraded and the proteolytic capacity of the cell. Together, the UPR and UPS function together to maintain proteostasis. The UPR is a conserved eukaryotic cell stress response system that is triggered by an accumulation of misfolded damaged proteins^37^. *P. falciparum* and other Apicomplexan parasites only have components of the translational arm of the UPR^38^. In response to artemisinin-mediated accumulation of misfolded proteins^39^, parasites activate the UPR via protein kinase 4 (PK4), a *P. falciparum* ortholog of protein kinase R (PKR)-like endoplasmic reticulum kinase (PERK)^8,40,41^. Under basal conditions, PK4 is held inactive by binding immunoglobulin protein (BiP)^40^. Accumulation of misfolded proteins in the endoplasmic reticulum lumen selectively titrates BiP away from PK4, releasing PK4 to phosphorylate its substrate, eukaryotic initiation factor 2, alpha subunit (eIF2α)^40^. Phosphorylated eIF2α (p-eIF2α) attenuates global protein translation^8,41^, allowing parasites to devote resources to mitigate existing damage by upregulating chaperones and proteasomes.

The misfolded proteins that trigger UPR activation must be degraded to resolve the UPR. In eukaryotes, the proteasome is responsible for the majority of protein degradation^42–44^. To be sent to the proteasome, damaged or misfolded proteins are generally tagged with several lysine^48^ (K48)- or lysine^11^ (K11)- linked ubiquitin moieties^45^. In *Plasmodium*, over 90% of ubiquitin linkages are proteasome targeting with 80% being K48-linkages and 13% being K11-linkages^46^. Furthermore, the proteasome has been shown to be essential for parasite survival in blood, liver, and mosquito stages^47,48^. The 26S proteasome, which mediates ubiquitin-dependent protein degradation, is a multi-subunit complex consisting of a 20S core particle (CP) capped by a 19S regulatory particle (RP)^49^. The 20S CP is composed of four heptameric rings: two α-rings that sandwich two β-rings. The catalytic β1, β2, and β5 subunits are responsible for protein cleavage and have caspase-like, trypsin-like, and chymotrypsin-like activity, respectively^50^. The 19S RP is composed of a lid and a base that mediates substrate recognition, deubiquitination, unfolding, and translocation into the 20S CP^49^. Prior to protein degradation, ubiquitin is removed from target substrates by 19S subunits that have deubiquitinase activity and by proteasome-associated deubiquitinases^49^. In addition to the 19S RP, the 20S CP can also be activated by PA28, which mediates ubiquitin-independent degradation of oxidized and unstructured proteins in mammals^51^. The 26S proteasome (19S RP complexed with the 20S CP) has been purified biochemically^52^ and the PA28–20S CP complex and 20S CP alone have been visualized by cryo-EM^53,54^, indicating that the 20S CP exists in multiple configurations in *P. falciparum*.

Previously, we reported that despite harboring Kelch13 C580Y, parasites with an additional mutation in the β2 proteasome catalytic subunit of C31Y or C31F display increased sensitivity to dihydroartemisinin (DHA), the active metabolite of all clinical artemisinins, and the related peroxide OZ439 (also known as artefenomel)^55^. This increase in sensitivity was not only observed at the early ring stage where artemisinin resistance is classically observed, but also throughout the asexual life cycle^55^. These data suggest that the proteasome is critical for parasites to survive artemisinins and acts in a manner distinct from Kelch13. We and others have shown that proteasome inhibitors synergize with DHA to potently kill artemisinin-resistant *P. falciparum* in vitro and in vivo^56,57^. Aside from DHA, proteasome inhibitors also synergized with distinct antimalarial compounds such as the peroxide OZ439, the deubiquitinase inhibitor b-AP15, and the redox inhibitor methylene blue, which are structurally diverse and possess distinct antimalarial modes of action^57^. Given the crucial role of proteasomes in restoring proteostasis, we were intrigued if the observed synergy was due to additional perturbation of proteostasis mechanisms by these synergistic compounds. To interrogate the role of proteostasis mechanisms in parasite artemisinin response and resistance, we examined UPR kinetics and proteasome activity in Kelch13 mutants and proteasome mutants. Our data show that Kelch13 WT and Kelch13 mutant parasites display distinct stage-dependent UPR kinetics. Importantly, early responses of hyperactivation and a concomitant unresolved UPR dictate eventual death in artemisinin-sensitive Kelch13 WT parasites. Finally, we show that a well-functioning proteasome promotes parasite survival to artemisinin, independent of the canonical K13-mediated resistance pathway.

## Results

### Antimalarial compounds synergistic with proteasome inhibitors disrupt proteostasis

**Figure 1:**
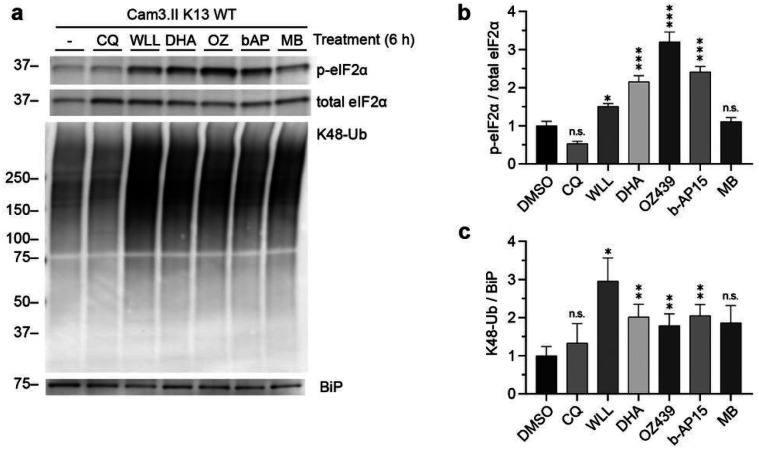
Antimalarial compounds synergistic with proteasome inhibitors disrupt proteostasis. **(a)** Cam3.II K13 WT parasites were synchronized to the trophozoite stage (26–30 hpi) and treated with DMSO, 50 μM chloroquine (CQ), 2.5 μM WLL, 50 nM dihydroartemisinin (DHA), 500 nM OZ439, 5 μM b-AP15, or 500 nM methylene blue (MB). All treatments were at 5x IC_50_ concentrations. Lysates were subject to Western blot and immunoblotted with antibodies against p-eIF2α, total eIF2α, K48-linked ubiquitin, and BiP. Shown is a representative blot from four independent experiments (see Supplementary Fig. 1 for replicates). (**b, c**) Densitometry analyses was performed using Image J to (**b**) assess UPR activation by normalizing p-eIF2α to total eIF2α and (**c**) assess proteasome inhibition by normalizing K48-Ub to the loading control BiP. Bar graphs indicate mean normalized integrated density ± S.E.M. Statistical significance was examined for each treatment against the DMSO control using a two-tailed paired *t*-test. *p < 0.05; **p < 0.01; ***p < 0.001; n.s. = not significant.

We previously showed that the *P. falciparum*-specific proteasome inhibitors WLL and WLW synergize with four of sixteen candidate and clinically used antimalarials^58^. The four synergistic compounds (DHA, OZ439, b-AP15, and methylene blue) are structurally diverse and have distinct modes of action. DHA and OZ439 non-specifically alkylate nearby proteins^5,6^, b-AP15 inhibits a proteasome-associated deubiquitinase^59^, and methylene blue interferes with redox homeostasis^60,61^. We were curious why these different classes of antimalarials were synergistic with proteasome inhibitors, and hypothesized that they may perturb proteostasis. To this end, Cam3.II Kelch13 WT parasites were synchronized to 26–30 hpi trophozoite stages and treated for 6 h with a 5x IC_50_ concentration of the proteasome inhibitor WLL^56^, the synergistic compounds DHA, OZ439, b-AP15, and methylene blue, the antagonistic compound chloroquine, or the vehicle control, DMSO. UPR activation was determined by levels of p-eIF2α, a marker of UPR activation, normalized to total eIF2α levels^62^. Proteasome dysfunction was determined by levels of K48-linked ubiquitination^63^ normalized to BiP, because in Cam3.II strain parasites BiP does not increase in response to DHA^36^. Treatment with the synergistic compounds DHA, OZ439, and b-AP15 all resulted in UPR activation with OZ439 resulting in the greatest UPR activation followed by DHA and b-AP15 treatment yielding similar levels of UPR activation (Fig. 1a, b, and **Supplementary Fig. 1**). These three compounds led to an accumulation of K48-linked ubiquitination, and the effect on ubiquitination from each of these compounds was similar (Fig. 1a, c, and **Supplementary Fig. 1**). Methylene blue, which was synergistic with proteasome inhibitors in ring stages but additive in trophozoite stages^58^, did not activate the UPR but led to a 2-fold increase in K48-linked ubiquitination, although this was not statistically significant (*p* = 0.2014; Fig. 1a–c and **Supplementary Fig. 1**). In contrast, the antagonistic compound chloroquine^58^, which inhibits heme detoxification^64^, did not alter levels of p-eIF2α or K48-linked ubiquitination relative to the DMSO-treated control (Fig. 1a–c and **Supplementary Fig. 1**). As a positive control for proteasome inhibition, parasites were treated with WLL. Indeed, WLL-treated parasites accumulated high levels of K48-linked ubiquitination (Fig. 1a, c, and **Supplementary Fig. 1**). A more moderate UPR activation was observed with WLL treatment, corroborating the primary effect on proteasome inhibition leading to the secondary effect of UPR activation^65,66^. Together, these data indicate that compounds that synergize with proteasome inhibitors to potently kill malaria parasites disrupt proteostasis, and suggest that the proteasome is important for parasite proteostasis restoration.

### Kelch13 WT and Kelch13 mutant parasites differentially regulate the UPR

**Figure 2:**
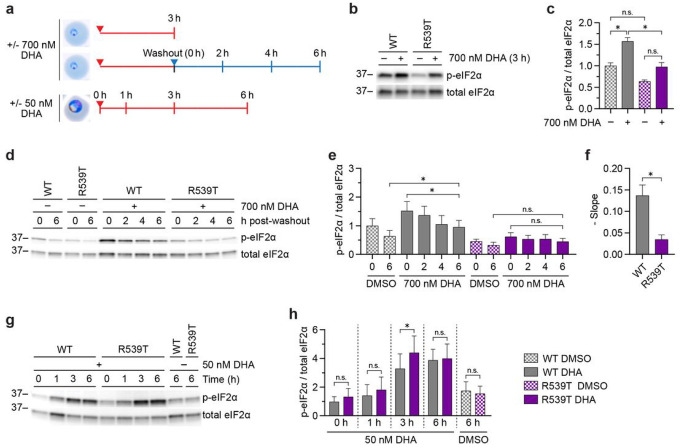
Kelch13 WT and Kelch13 mutant parasites differentially regulate the UPR. **(a)** UPR activation was assessed in WT and R539T parasites under the depicted conditions. Red arrows indicate addition of drug and red lines indicate drug treatment. The blue triangle indicates time of drug washout and the blue line indicates time after washout. (**b**) WT and R539T parasites were synchronized to 0–3 hpi rings and treated with DMSO or 700 nM DHA for 3 h, then lysates were subject to Western blot and immunoblotted with antibodies against p-eIF2α and eIF2α. Shown is a representative blot from three independent experiments (see Supplementary Fig. 2a for replicates). (**c**) Densitometry analyses was performed using Image J and UPR activation determined as described in Fig.1. (**d**) WT and R539T parasites were synchronized to 0–3 hpi rings and treated with DMSO or 700 nM DHA for 3 h. Then drug was washed off and parasites were harvested at the indicated times to monitor UPR resolution. Western blot was performed as described in (b). Representative blot shown from three independent experiments (see Supplementary Fig. 2b for replicates). (**e**) Densitometry and determination of UPR activation performed as described in (c). (**f**) Rate of de-phosphorylation of eIF2α following drug removal was calculated over time and the mean negative slope ± S.E.M. was plotted. (**g**) WT and R539T parasites were synchronized to 26–30 hpi trophozoites, then treated with DMSO or 50 nM DHA for the indicated times. Western blot was performed as described in (b). Representative blot shown from four independent experiments (see Supplementary Fig. 2f for replicates). (**h**) Densitometry and determination of UPR activation performed as described in (c). Bar graphs indicate mean normalized integrated density ± S.E.M. from at least three independent experiments. Statistical significance was examined for the indicated comparisons using a two-tailed paired *t*-test. *p < 0.05; n.s. = not significant.

The UPR is an exquisitely well-regulated process, and we were interested in understanding the kinetics of UPR activation and resolution in artemisinin-sensitive and artemisinin-resistant parasites. To do so, Cam3.II Kelch13 WT (hereon referred to as WT; Table 1) and Cam3.II Kelch13 R539T parasites (hereon referred to as R539T; Table 1) were tightly synchronized to 0–3 hpi rings and treated with the physiologically-relevant concentration of 700 nM DHA for 3 h, mimicking conditions of the RSA used to delineate artemisinin resistance in vitro^28^ (Fig. 2a, **top and middle panels**). In response to DHA, levels of p-eIF2α increased 1.5-fold in both parasites. However, only DHA-treated WT parasites had significantly higher levels of p-eIF2α compared to mock-treated controls and relative to the DHA-treated R539T mutant (Fig. 2b, c, **Supplementary Fig. 2a**).

Next, UPR resolution was monitored in these parasites following drug removal. Levels of p-eIF2α declined over time in both parasites following DHA washout (Fig. 2d, e, f, **Supplementary Fig. 2b**). However, by 6 h post washout, levels of p-eIF2α in WT parasites remained elevated relative to the mock-treated control (Fig. 2d and e), suggesting that these parasites were unable to resolve the UPR and remained in a state of stress. In contrast, at 6 h post-washout, levels of p-eIF2α in R539T parasites returned to basal levels (Fig. 2d and e). Cam3.II Kelch13 C580Y (hereon referred to as C580Y; Table 1) parasites, which display an intermediate RSA value between WT and R539T parasites^55,67^, displayed intermediate UPR activation and resolution as measured by the rate of p-eIF2α de-phosphorylation following DHA removal (**Supplementary Fig. 2c-e**). Interestingly, C580Y parasites with an additional β2 C31Y mutation, which sensitized parasites to DHA^68^, also had elevated levels of p-eIF2α at 6 h post-washout compared to mock-treated counterparts (**Supplementary Fig. 2c-e**). Together, the data suggest that parasites sensitized to DHA are unable to resolve DHA-mediated UPR activation despite removal of the drug.

To determine if these phenotypes would be maintained at the trophozoite stage, UPR activation was monitored in WT and R539T parasites synchronized to 26–30 hpi trophozoites (Fig. 2A, **bottom panel**). Upon treatment with 50 nM DHA, a 5x IC_50_ concentration, the UPR was activated in both parasites in a time-dependent manner. Of note, levels of p-eIF2α were significantly higher at 3 h in R539T *vs*. WT parasites (Fig. 2g, h, and **Supplementary Fig. 2f**), suggesting a more robust UPR activation in the R539T mutant. By 6 h post-treatment, levels of p-eIF2α were similar between the parasites examined (Fig. 2g, h, and **Supplementary Fig. 2f**). These data show that the kinetics of UPR activation and resolution are dependent on both Kelch13 genotype and the parasite stage during its intraerythrocytic development cycle.

### Peroxides DHA and OZ439 inhibit parasite proteasome activity

Previously it was shown that DHA inhibits β5 proteasome catalytic activity in artemisinin-sensitive Kelch13 WT parasites and leads to an accumulation of ubiquitinated proteins^8,69^. Since artemisinin-resistant and Kelch13 mutant parasites have been shown to express higher levels of proteasome subunits^35,36^, we sought to determine whether Kelch13 mutations impacted DHA-mediated proteasome inhibition. Although the β5 catalytic activity is responsible for the majority of protein degradation, we were also interested in the effect of DHA on the other two catalytic subunits of the proteasome as they play a role in protein degradation, in addition to the effect of the related peroxide OZ439 on proteasome catalytic activity. Proteasome activity in DHA-treated WT, R539T, and C580Y parasites was examined using two orthogonal approaches. For both approaches, trophozoite stages were assayed since the UPS is upregulated at the trophozoite stage^46,70^ and artemisinin treatment does not produce a detectable increase in ubiquitination at the early ring stage^69^. In the first approach, proteasome subunit catalytic activity was examined in DHA-treated and OZ439-treated trophozoites using the fluorogenic substrates Ac-nLPnLD-AMC, Ac-RLR-AMC, or Suc-LLVY-AMC to assess caspase-like, trypsin-like, and chymotrypsin-like activity, respectively^71^. Though these fluorogenic substrates can react with other proteases in the parasites, for simplicity we will refer to caspase-like activity as β1 activity, trypsin-like activity as β2 activity, and chymotrypsin-like activity as β5 activity. WLL, a *P. falciparum*-selective proteasome inhibitor with activity against β2 and β5 active sites^56^ was used as a positive control for inhibiting these two catalytic sites. No known inhibitor of plasmodial β1 exists, though high concentrations of WLL have been shown to moderately inhibit plasmodial β1 activity^56^.

**Figure 3:**
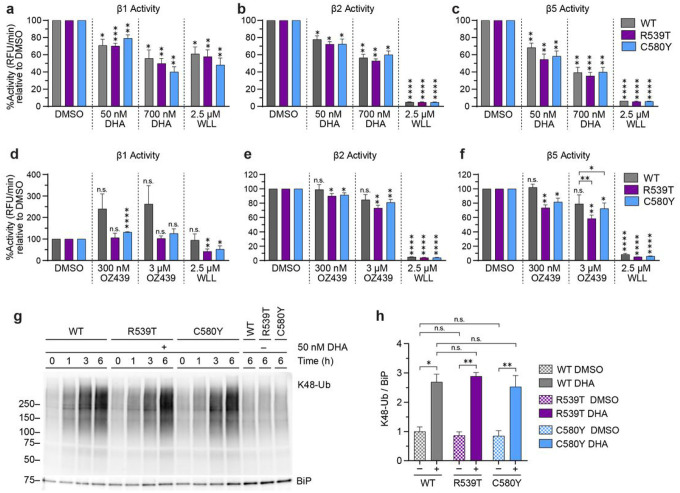
Peroxides DHA and OZ439 inhibit parasite proteasome activity. **(a-c)** WT, R539T, and C580Y parasites were synchronized to 26–30 hpi trophozoites and treated with DMSO, 50 nM DHA, 700 nM DHA, or 2.5 μM WLL for 3 h. Then, protein was harvested and incubated with (**a**) Ac-nLPnLD-AMC, (**b**) Ac-RLR-AMC, or (**c**) Suc-LLVY-AMC to assess β1, β2, and β5 activity, respectively. Fluorescence was plotted over time and % activity was quantified by calculating the slope of the line and normalizing to the slope of DMSO-treated parasites. Bar graphs indicate mean % activity ± S.E.M. A two-tailed Student’s *t*-test was performed between DMSO and drug-treatment counterparts, and statistical significance is indicated above the bars as vertical asterisks. Comparisons were also performed between Kelch13 WT and Kelch13 mutants for each treatment condition, but no significant difference was found (only significant comparisons between WT and mutant parasites are denoted here). (**d-f**) Parasites were synchronized as described above but treated with DMSO, 300 nM OZ439, 3 μM OZ439, or 2.5 μM WLL for 3h. Then, protein was harvested and proteasome activity was assessed as described above. (**g**) WT, R539T, and C580Y parasites were treated with DMSO or 50 nM DHA for the indicated times. Lysates were subjected to Western blot and immunoblotted with antibodies against K48-linked ubiquitin and BiP. Shown is a representative blot of four independent experiments. (see Supplementary Fig. 3 for replicates). (**h**) Densitometry analyses was performed with Image J and levels of K48-linked ubiquitination was normalized to the loading control BiP. Bar graphs indicate mean normalized integrated density ± S.E.M. A two-tailed paired *t*-test was performed for the indicated comparisons. *p < 0.05; **p < 0.01; ***p < 0.001; ****p < 0.0001; n.s. = not significant.

DHA inhibited β1 (Fig. 3a), β2 (Fig. 3b), and β5 (Fig. 3c) activity in WT, R539T, and C580Y trophozoites in a statistically significant and concentration-dependent manner. β1 and β2 activity were inhibited by approximately 30% and 40% following treatment with 50 nM DHA and 700 nM DHA, respectively (Fig. 3a and b). β5 activity was inhibited to the greatest extent, with approximately 40% and 60% inhibition upon treatment with 50 nM DHA and 700 nM DHA, respectively (Fig. 3c). In addition to comparing treated to untreated counterparts as detailed above, we also tested for differences in DHA-mediated inhibition depending on Kelch13 genotype but no significant difference in catalytic inhibition was detected between Kelch13 WT and Kelch13 mutant parasites. The DHA-related peroxide OZ439 did not inhibit β1 activity of proteasomes isolated from all tested parasite strains (Fig. 3d). Intriguingly, OZ439 modestly inhibited β2 activity (10–15% inhibition) of proteasomes derived from R539T and C580Y but not WT parasites (Fig. 3e). In addition, OZ439 selectively inhibited β5 activity (approximately 25% inhibition) of proteasomes derived from Kelch13 mutants, which was determined to be statistically significant at the peak plasma concentration of 3 μM OZ439^72,73^ (Fig. 3f).

Although fluorogenic substrate assays accurately determine proteasome catalytic activity, these assays are unable to measure proteasome-mediated protein degradation. Thus, in a second approach to measure proteasome activity, we examined the accumulation of K48-linked ubiquitination, which is a hallmark of proteasome dysfunction. Synchronized WT, R539T, and C580Y strain parasites at the 26–30 hpi trophozoite stages were treated with 50 nM DHA for up to 6 h, then lysates were examined for protein ubiquitination. In response to DHA, all parasites showed a statistically significant accumulation of K48-linked ubiquitination in a time-dependent manner (Fig. 3g, h, **Supplementary Fig. 3a**). At each timepoint, levels of ubiquitination was similar across all parasites tested regardless of Kelch13 genotype (**Supplementary Fig. 3b**), reflecting results obtained from proteasome catalytic activity assays. Collectively, these data show that DHA equally inhibits proteasomes from WT, R539T, and C580Y. In contrast, OZ439 selectively inhibits the β5 catalytic activity of proteasomes derived from R539T and C580Y parasites.

### Mutations in 19S proteasome subunits increase parasite susceptibility to DHA

**Figure 4:**
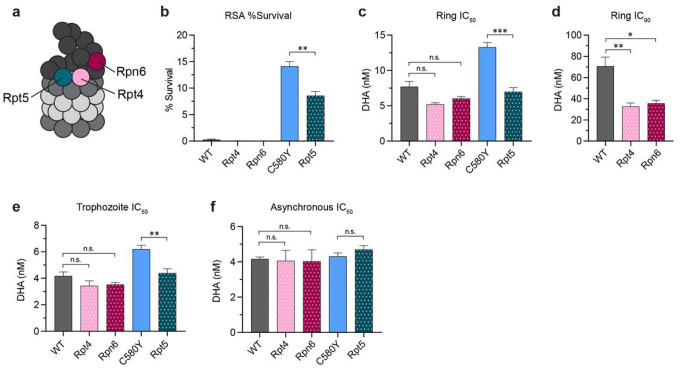
19S proteasome mutants display increased susceptibility to DHA. **(a)** Diagram of 26S proteasome showing placement of Rpt5, Rpn6, and Rpn4 within the multisubunit complex. (**b-d**) WT, Rpt4, Rpn6, C580Y, and Rpt5 parasites were tightly synchronized to 0–3 hpi rings, then exposed to a range of DHA concentrations starting at 1400 nM DHA for 3 h. DHA was washed off, and parasitemia was assessed 66 h later. (b) % survival was calculated by dividing the parasitemia of parasites treated with 700 nM DHA by the parasitemia of mock-treated parasites. (c) ring-stage IC_50_ and (d) ring-stage IC_90_ values were determined using non-linear regression analysis. (e) Parasites were tightly synchronized to 26–30 hpi trophozoites, then exposed to a range of DHA concentrations for 3 h. DHA was washed off, parasitemia assessed 66 h later, and trophozoite-stage IC_50_ values determined as above. (f) Dose response assays with DHA were conducted on asynchronous parasites, parasitemia assessed 72 h later, and IC_50_ values determined as above. Bar graphs show mean IC_50_ or IC_90_ values ± S.E.M., determined from at least four independent experiments. Statistical significance was examined for each proteasome mutant against the cognate parental strain using a Mann-Whitney *U* test. *p < 0.05; **p < 0.01; ***p < 0.001; n.s. = not significant.

Previously, we reported that an additional mutation in the 20S β2 proteasome subunit at either C31Y or C31F in the context of a C580Y background increased parasite susceptibility to DHA^55^. These parasites were generated via in vitro selection studies with the *P. falciparum*-specific proteasome inhibitor WLW^58^. In the same WLW selection study, three 19S proteasome mutants were also selected for in the Cam3.II background: Cam3.II Kelch13 WT Rpt4 E380*, Cam3.II Kelch13 WT Rpn6 E266K, and Cam3.II Kelch13 C580Y Rpt5 G319S^58^ (hereon referred to as Rpt4, Rpn6, and Rpt5, respectively; Table 1). Rpt4 and Rpt5 are ATPase subunits in the 19S RP base, which mediate gate opening to allow substrates into the 20S^49^. Rpt4 is also in contact with the 19S lid^74^. Rpn6 acts as a scaffolding protein that stabilizes the interaction between the 19S and 20S^75^ (Fig. 4a). The 19S RP is important for regulating protein processing prior to proteolytic degradation within the 20S CP chamber^49^. Thus, we were interested in determining if mutations in 19S subunits in the context of a C580Y background would compromise parasite resistance to DHA and if such mutations evolved on a WT background would further hypersensitize parasites to DHA.

To this end, dose response assays were performed using early ring (0–3 hpi), trophozoite (26–30 hpi), and asynchronous cultures (**Supplementary Fig. 4**). At 0–3 hpi, all parasites with a Kelch13 WT background displayed RSA values < 1% (Fig. 4b). While WT, Rpt4, and Rpn6 all had similar IC_50_ values (WT = 7.7 nM; Rpt4 = 5.2 nM; Rpn6 = 6.0 nM; Fig. 4c), the Rpt4 and Rpn6 mutants had 2-fold lower IC_90_ values than their parent (WT = 70.7 nM; Rpt4 = 32.7 nM; Rpn6 = 35.6 nM; Fig. 4d) and had correspondingly steeper dose response curves (**Supplementary Fig. 4a**). Relative to its parent C580Y, the Rpt5 mutant had a 1.5-fold lower RSA value (C580Y = 14.1%; Rpt5 = 8.6%; Fig. 4b) and a 2-fold lower IC_50_ value (C580Y = 13.3 nM; Rpt5 = 7.0 nM; Fig. 4c).

At the trophozoite stage, WT, Rpt4, and Rpn6 parasites had similar dose response curves (**Supplementary Fig. 4b**) and IC_50_ values of approximately 4 nM (Fig. 4e). Relative to C580Y with an IC_50_ of 6.2 nM, the Rpt5 mutant again displayed increased sensitivity to DHA at the trophozoite stage with an IC_50_ of 4.4 nM (Fig. 4e). In asynchronous cultures, all tested parasites displayed similar dose response curves (**Supplementary Fig. 4c**) and IC_50_ values of approximately 4 nM (Fig. 4f). These data demonstrate that indeed, mutations in 19S subunits increase parasite susceptibility to DHA and can even hypersensitize parasites to DHA when 19S mutations occur on a Kelch13 WT background.

### Parasites with increased peroxide susceptibility have impaired proteasome-mediated protein degradation

**Figure 5:**
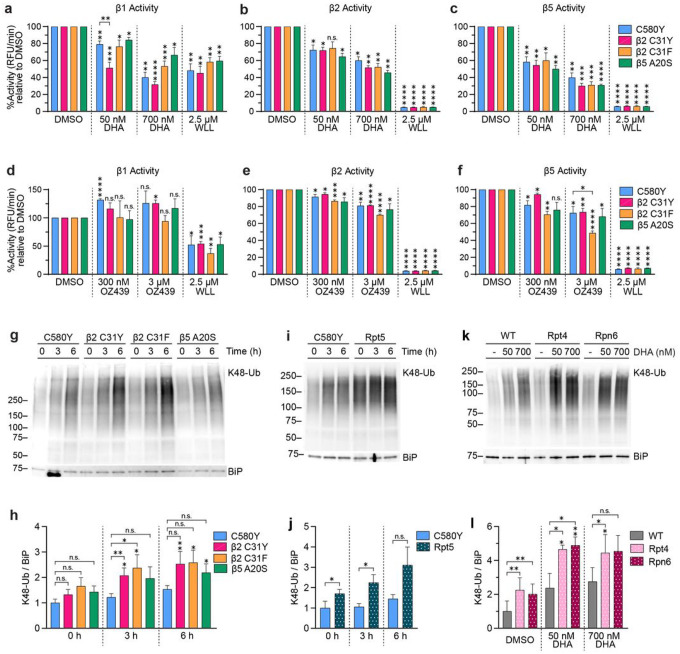
Proteolytic activity of proteasome mutants. **(a-c)** Trophozoites were treated with as indicated for 3 h. Protein lysates were incubated with (**a**) Ac-nLPnLD-AMC, (**b**) Ac-RLR-AMC, or (**c**) Suc-LLVY-AMC to assess β1, β2, and β5 activity, respectively. Proteasome activity was assessed as described in Fig. 3. A two-tailed Student’s *t*-test was performed between DMSO and drug-treated counterparts, indicated above the bars as vertical asterisks. Comparisons were also performed between proteasome mutants and C580Y and only significant comparisons are denoted with brackets. (**d-f**) Parasites were synchronized as described above but treated with DMSO, 300 nM OZ439, 3 μM OZ439, or 2.5 μM WLL for 3h. Then, protein was harvested and proteasome activity assessed as described. (**g**) Trophozoites from the indicated parasites were treated with 50 nM DHA for 0, 3, and 6 h. Lysates were subjected to Western blot and immunoblotted with antibodies against K48-linked ubiquitin and BiP. Representative blot shown out of five independent experiments. (see Supplementary Fig. 5a for replicates). (**h**) Densitometry analyses was performed using Image J. Levels of K48-linked ubiquitination was normalized to BiP. Bar graph indicates mean normalized integrated density ± S.E.M. A one-tailed paired *t*-test was performed to compare treated and untreated counterparts, indicated above the bars as vertical asterisks. A one-tailed paired *t*-test was also performed between proteasome mutants and C580Y, indicated by brackets. (**i**) 26–30 hpi trophozoites derived from C580Y and Rpt5 parasites were treated with 50 nM DHA for indicated times. Western blot performed as described in (g). Shown is a representative blot out of four independent experiments. (see Supplementary Fig. 5b for replicates). (**j**) Densitometry analyses and quantitation performed as in (h). (**k**) WT, Rpt4, and Rpn6 trophozoites were treated with DMSO, 50 nM, or 700 nM DHA for 3 h. Western blot performed as described in (g). Shown is a representative blot out of three independent experiments. (see Supplementary Fig. 5c for replicates). (**l**) Densitometry analyses and quantitation performed as in (h). *p < 0.05; **p < 0.01; ***p< 0.001; ****p < 0.0001; n.s. = not significant.

Given the increased peroxide susceptibility of parasites harboring 19S or 20S mutations, we hypothesized that the proteasome is essential for parasite survival in the face of artemisinin and similar compounds, and that the observed sensitivity of proteasome mutants to peroxides is due to a dysfunction in proteasome-mediated protein degradation. To test this hypothesis, we examined the proteasome catalytic activity of DHA- and OZ439-treated 26–30 hpi trophozoites derived from the C580Y parental strain and cognate 20S CP mutants: Cam3.II Kelch13 C580Y β2 C31Y, Cam3.II Kelch13 C580Y β2 C31F, and Cam3.II Kelch13 C580Y β5 A20S (hereon referred to as β2 C31Y, β2 C31F, and β5 A20S, respectively; Table 1). Treatment with DHA significantly inhibited β1 (Fig. 5a), β2 (Fig. 5b), and β5 (Fig. 5c) activity in all parasites tested in a concentration-dependent manner. In addition, relative to its parent, the β2 C31Y mutant displayed greater inhibition of β1 activity upon treatment with 50 nM DHA (C580Y = 20% inhibition; β2 C31Y = 49% inhibition), but no other significant difference was observed between DHA-treated parent and proteasome mutant parasites. OZ439 did not inhibit β1 activity (Fig. 5d), but did inhibit β2 and β5 activities in all parasites tested (Fig. 5e, f). Note that at 3 μM OZ439, the β5 catalytic site of the β2 C31F mutant was significantly more inhibited compared to that of the parental C580Y parasite (C580Y = 28% inhibition; β2 C31F = 52% inhibition; Fig. 5f). No other significant difference in catalytic inhibition was observed between parental and 20S proteasome mutants treated with OZ439.

Next, inhibition of proteasome-mediated protein degradation was evaluated in 20S mutant (Fig. 5g, h) and 19S mutant parasites (Fig. 5i–l) by assessing accumulation of K48-linked ubiquitination. DHA-treated C580Y and β2 mutants led to significantly increased K48-linked ubiquitination compared to mock-treated parasites (Fig. 5g, h, and **Supplementary Fig. 5a**). In addition, relative to their parent C580Y, both β2 C31Y and β2 C31F mutants had 1.5- to 2-fold higher levels of K48-linked ubiquitination in response to 3 h treatment of DHA (Fig. 5g, h, and **Supplementary Fig. 5a**). The β5 A20S mutant, which did not display altered sensitivity to DHA or OZ439^55^, had minor and statistically insignificant increases in ubiquitination (Fig. 5g, h, and **Supplementary Fig. 5a**). Derived on a genetic background expressing Kelch13 C580Y, Rpt5 mutants displayed a statistically significant 2-fold increase in K48 ubiquitination compared to the parental strain at basal levels without any drug treatment and at 3h DHA treatment (Fig. 5i, j, and **Supplementary Fig. 5b**). For the 19S mutants that were derived on a Kelch13 WT background, Rpt4 and Rpn6 mutants also showed a statistically significant 2-fold increase in ubiquitination compared to WT at basal levels and upon drug treatment (Fig. 5k, l, and **Supplementary Fig. 5c**). In addition, DHA-treated Rpt4 and Rpn6 mutants accumulated significantly more ubiquitination compared to mock-treated counterparts (Fig. 5k, l, and **Supplementary Fig. 5c**). Since we observed that the UPR was differentially activated in Kelch13 WT *vs*. Kelch13 mutant parasites, we were also interested in determining UPR activation kinetics in proteasome mutants. However, no significant difference in UPR activation was observed between parental and proteasome mutant parasites at the early ring stage (**Supplementary Fig. 2c-e**) or trophozoite stage (**Supplementary Fig. S6**). We note that in trophozoite stages, C580Y parental strain as well as β2 C31Y, β2 C31F, β5 A20S, and Rpt5 proteasome mutants significantly induced UPR activation compared to untreated counterparts, albeit the level of UPR activation was similar for parent and proteasome mutants (**Supplementary Fig. S6**). Collectively, these data indicate that a defect in proteasome-mediated protein degradation underlies the heightened sensitivity of proteasome mutants to peroxides, and that this defect is not mediated by increased inhibition of proteasome catalytic subunits.

### The β2 C31Y proteasome mutant is sensitized to proteasome-related inhibitors

**Figure 6:**
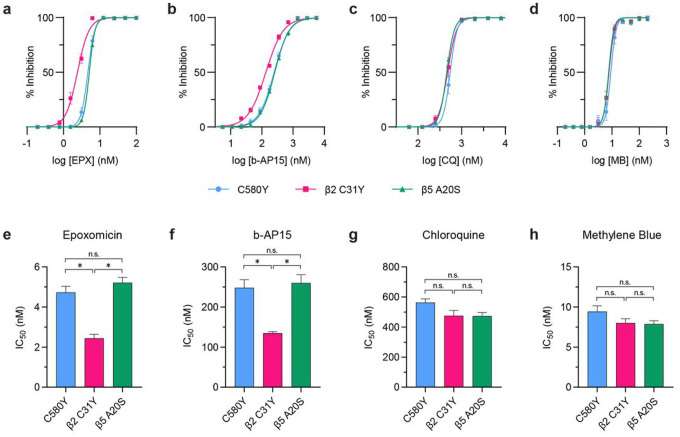
The β2 C31Y mutant shows increased susceptibility to proteasome-related inhibitors. **(a-d)** 72-hour dose response assays were conducted on asynchronous C580Y, β2 C31Y, and β5 A20S parasites with (**a**) epoxomicin (EPX), (**b**) b-AP15, (**c**) chloroquine (CQ), and (**d**) methylene blue (MB). Data points on the graph indicate mean % inhibition ± S.E.M. (**e-h**) IC_50_ values were calculated using non-linear regression analysis and plotted as bar graphs. Bar graphs depict mean IC_50_ values ± S.E.M. Data shown is from four independent biological replicates. Statistical significance was determined using a Mann-Whitney *U* test. *p < 0.05; n.s. = not significant.

Given our observation that β2 mutants exhibited proteasome dysfunction compared to the parental C580Y as well as the β5 A20S mutant, we reasoned that in addition to DHA and OZ439, β2 mutants should also selectively display increased susceptibility to compounds that inhibit proteasome-mediated protein degradation. To test this hypothesis, C580Y, β2 C31Y, and β5 A20S strain parasites were subjected to dose response assays with epoxomicin, a non-parasite selective inhibitor of the proteasome^76^, and b-AP15, an inhibitor of a proteasome-associated deubiquitinase^59^. As negative controls, we included chloroquine and methylene blue, both of whose mechanisms of action are unrelated to the proteasome^60,61,64,77^. The C580Y parent and the β5 A20S mutant, which have similar sensitivity profiles to DHA and OZ439^55^, displayed almost identical dose-response curves in response to epoxomicin (Fig. 6a), b-AP15 (Fig. 6b), chloroquine (Fig. 6c), and methylene blue (Fig. 6d). Accordingly, C580Y and β5 A20S also had similar IC_50_ values of 4.7 nM and 5.2 nM for epoxomicin (Fig. 6e), 248.0 nM and 259.9 nM for b-AP15 (Fig. 6f), 561.9 nM and 472.6 nM for chloroquine (Fig. 6g), and 9.4 nM and 7.9 nM for methylene blue (Fig. 6h). In contrast, the β2 C31Y mutant displayed a 2-fold increased susceptibility to epoxomicin (C580Y = 4.7 nM; β2 C31Y = 2.4 nM; Fig. 6e) and b-AP15 (C580Y = 248.0 nM; β2 C31Y = 134.6 nM; Fig. 6f), but not to chloroquine (C580Y = 561.9 nM; β2 C31Y = 474.6 nM; Fig. 6g) or methylene blue (C580Y = 9.4 nM; β2 C31Y = 8.0 nM; Fig. 6h).

Dose response curves of slow growing parasites may be shifted left due to a defect in parasite fitness that is unrelated to parasite susceptibility to a particular compound. Given that the β2 C31Y mutant and its parent had similar dose response curves for chloroquine and methylene blue, it is unlikely that the increased susceptibility of this parasite to DHA, OZ439, epoxomicin, and b-AP15 is due to a fitness cost. However, to rule out this possibility, parasite fitness competition assays were conducted with C580Y, β2 C31Y, β2 C31F, and β5 A20S parasites. No significant difference was found between C580Y and the 20S proteasome mutants examined (**Supplementary Fig. 7**). Thus, these data demonstrate that the β2 C31Y mutant is selectively sensitive to compounds that target the proteasome.

## Discussion

As the prevalence of artemisinin resistance continues to rise, it becomes increasingly urgent to delineate a mechanism of resistance to inform future drug discovery and implementation of antimalarial combination therapies. In addition to the widespread artemisinin resistance in the Southeast Asian region, recent reports of Kelch13-mediated artemisinin resistance in Rwanda and Uganda detected in the past three years is of particular concern^14,19,25 26,27,78^. We have previously shown that proteasome inhibitors effectively kill artemisinin-resistant parasites and strongly synergize with DHA^57,79^. In addition, parasites moderately resistant to proteasome inhibitors are sensitized to DHA^68^. Importantly, proteasome inhibitors are effective against Ugandan parasite isolates^80^.

The proteasome is intimately involved in the UPR and protein degradation, two pillars of proteostasis. In a well-functioning cell, UPR activation will lead to upregulation of proteasome-mediated protein degradation, and inhibition of proteasome-mediated protein degradation will lead to UPR activation^65,66^. Exploration of the kinetics of UPR activation and resolution as well as proteasome activity in isogenic parasites only differing in the loci of Kelch13 or proteasome subunits yielded some surprising results.

Firstly, our data confirmed our hypothesis that antimalarials that synergize with proteasome inhibitors such as DHA, OZ439, and b-AP15 perturb proteostasis by upregulating the UPR and inhibiting proteasome-mediated protein degradation. In contrast, antimalarials that antagonize with proteasome inhibitors such as chloroquine had no effect on these measurements of proteostasis perturbations. Interestingly, methylene blue was additive with proteasome inhibitors, and had an intermediate increase in UPR activation and ubiquitination. These data suggest that directly interfering with proteostasis mechanisms is a promising antimalarial therapeutic strategy.

Secondly, we found that early parasite responses to DHA dictate eventual survival outcomes. Transcriptomics and proteomics data point to a role for Kelch13 mutants in broadly enhancing the parasite’s stress response^35,36^. However, the molecular stress response pathways involved, and a well-defined mechanism of resistance have not been elucidated. Here we show that artemisinin-sensitive Kelch13 WT parasites hyperactivate the UPR at early ring stages, indicating that these parasites are either (1) experiencing increased levels of stress and/or (2) the UPR is dysfunctionally regulated. Mislocalization studies suggest Kelch13 mutant parasites have reduced hemoglobin uptake and digestion^33,34^, and it is hypothesized that as a consequence these parasites have reduced artemisinin activation. However, the role of Kelch13 in hemoglobin uptake appears to be restricted to the ring stage^33^. Accordingly, it would be expected that the misfolded protein load in Kelch13 mutants would be lower and less prone to trigger the UPR at the ring stage. This hypothesis is consistent with our observations that early ring stage R539T parasites display little UPR activation in response to DHA and are also able to completely resolve the UPR following drug removal. In contrast, DHA-treated WT early rings display robust UPR activation and are unable to completely resolve the UPR, as seen by residual eIF2α phosphorylation 6 h after DHA removal. This is consistent with findings that following a 3 h pulse with 700 nM DHA on ring-stage parasites, R539T parasites begin to resume protein turnover as early as 9 h after drug removal while WT parasites do not, and the differences become more pronounced at 15 h post-drug withdrawal^34^. However, at the trophozoite stage, where hemoglobin digestion is increased^81^ and Kelch13 is not involved in hemoglobin uptake^33^, we found that the UPR was activated earlier in DHA-treated R539T parasites. Activating the UPR more quickly while in the trophozoite stages could be advantageous to Kelch13 mutants, giving them a jumpstart on mitigating protein damage, given that metabolic processes and protein abundance is greatly increased during these stages compared to ring stages^34^. However, a direct molecular link between Kelch13 and the UPR remains to be identified and are addressed in ongoing studies.

Previous studies demonstrate conflicting data regarding UPR activation in the early ring stages of Kelch13 WT *vs*. Kelch13 mutants. Consistent with what we observed, Dd2 Kelch13 WT 0–3 hpi rings treated with 700 nM DHA for 15 min displayed more robust UPR activation than Dd2 Kelch13 C580Y 0–3 hpi rings^41^. However, the authors observed that Kelch13 mutants displayed elevated basal UPR activation, which is in contrast to our observation^41^. This disparity could be attributed to differences in the genetic backgrounds of the parasites examined. Of note, Dd2 was adapted to the laboratory in the 1970s prior to widespread artemisinin usage, while Cam3.II was adapted in 2010s and originated from an artemisinin-resistant isolate. In a separate study, it was observed that relative to Cam3.II Kelch13 WT 0–8 hpi rings, Cam3.II Kelch13 R539T parasites had elevated UPR activation under basal conditions and in response to a 3 h treatment with 700 nM DHA^34^. It is possible that differences between early- and mid-ring stages could explain discrepancies between these data, and the less tightly synchronized rings in^34^ could be behaving more similarly to the trophozoite stage parasites in our study.

Isogenic Kelch13 mutant *vs*. Kelch13 WT parasites^36^ and artemisinin-resistant clinical isolates^35^ have been shown to have increased levels of proteasome subunits by transcriptomics and proteomics. However, we observed no noticeable difference in proteasome activity between isogenic Kelch13 WT *vs*. Kelch13 mutants at basal levels or when DHA-treated when we assessed model substrate cleavage as well as cellular protein degradation. Since the proteasome is a multi-subunit complex with particular stoichiometry and assembly of subunits, upregulation of some proteasome subunits may be insufficient to modulate proteasome activity. It is also possible that the assays used here are unable to detect slight differences in proteasome activity which may be biologically relevant. Collectively, these data suggest that Kelch13 does not mediate artemisinin resistance by modulating proteasome activity but rather by modulating UPR activation and resolution. It was recently reported that Kelch13 mutant parasites undergo higher levels of autophagy than Kelch13 WT parasites under basal conditions^82^, which would aid in disposing of damaged proteins thus complementing any deficiencies in proteasome-mediated protein degradation.

Yet, the proteasome may play a critical role in non-Kelch13-mediated artemisinin response. The third major finding of our study is that parasite susceptibility to DHA, mediated by mutations in the proteasome, correlated with a dysfunction in proteasome-mediated protein degradation. Previous studies showed that upon artemisinin treatment, the artemisinin-sensitive parasites 3D7 and PL2 (artemisinin-sensitive; Kelch13 WT) had a 2-fold increase in ubiquitination while the artemisinin-resistant PL7 strain (artemisinin-resistant; Kelch13 mutant) only accumulated ~1.2-fold increased ubiquitination^69^. Note that none of these three strains are isogenic, and there are multiple genetic differences between 3D7, PL2, and PL7, including at known drug resistance modulators such as *P. falciparum* multidrug resistance protein 1 (PfMDR1), *P. falciparum* multidrug resistance protein 2 (PfMDR2), and *P. falciparum* chloroquine resistance transporter (PfCRT)^69^. In our study, we corroborate these earlier data and show that parasites susceptible to DHA and isogenic except for mutations in proteasome subunits display increased ubiquitination. Not all proteasome mutations and resultant proteasome dysfunction affect DHA susceptibility equally across asexual blood stages. For example, while all 19S and β2 mutants tested displayed a defect in proteasome-mediated protein degradation, 19S mutants only displayed increased susceptibility to DHA in synchronized cultures, whereas the β2 proteasome mutants displayed increased sensitivity at ring, trophozoite, and asynchronous stages^55^. These data could indicate that the 20S plays an outsized role in parasite artemisinin response. Perhaps in addition to the 20S-19S complex, the 20S-PA28 complex contributes to resolving artemisinin-mediated protein damage. This is supported by previous findings that 3D7 parasites in which PA28 is knocked out display a 2-fold lower DHA IC_50_ values at the early ring stage^54^.

Although Rpt5, Rpt4, Rpn6, and β2 proteasome mutants showed increased ubiquitinated polypeptides in response to DHA compared to parental strains, these differences were not detected when we assayed for proteasome catalytic activity as measured by cleavage of fluorogenic peptidyl model substrates. One reason for this discrepancy is that the fluorogenic substrates can freely diffuse into the 20S CP without processing by the 19S RP, whereas detection of K48-linked ubiquitinated proteins assesses the ability of the 26S proteasome as a whole to process and degrade proteins. Interestingly, peptidyl substrate cleavage showed that at peak plasma concentrations, OZ439 significantly inhibits the β5 activity of R539T and C580Y proteasomes but does not inhibit WT proteasomes. This could explain why these artemisinin-resistant parasite strains do not exhibit cross-resistance to OZ439^83,84^. OZ439 also inhibited the β5 catalytic activity of β2 C31F significantly more than in the parental C580Y strain. These results are in concordance with our previous data showing that β2 C31F showed the greatest decrease in RSA values in response to OZ439^68^.

It remains unknown to what degree the proteasome mutations tested here affect proteasome activity physiologically. Based on the cryo-EM structure of the *P. falciparum* 20S proteasome, β2 C31Y and β2 C31F were mapped near the S1 binding pocket of the β2 active site and were predicted to impair WLW binding via steric hinderance^58^. The Rpt4 E380* and Rpn6 E266K mutations fall outside of conserved domains, while G319S is located within the AAA domain of Rpt5 (**Supplementary Fig. 8**). This could indicate the Rpt5 mutation is more detrimental to proteasome activity than Rpt4 and Rpn6 mutations. Consistent with this hypothesis, the Rpt5 mutant displays increased sensitivity to DHA at ring and trophozoite stages in comparison to the Rpt4 and Rpn6 mutants, which were only sensitized at the ring stage. However, without the generation of transgenic parasites, the degree of DHA sensitization conferred by particular proteasome mutations and the influence of Kelch13 cannot be determined.

In summary, the data presented here indicate that (1) antimalarial compounds that synergize with proteasome inhibitors perturb parasite proteostasis, (2) early parasite UPR signaling in response to DHA dictate eventual survival outcomes, and (3) parasite susceptibility to DHA correlates with a dysfunction in proteasome-mediated protein degradation. We show here and previously that chemical inhibition of the proteasome and mutations in the proteasome increase parasite susceptibility to DHA regardless of Kelch13 genotype^57,68^, highlighting the crucial role of the proteasome in parasite survival to artemisinin. These data point to the UPR and UPS, two pillars of proteostasis, as pathways that can be targeted to overcome existing artemisinin resistance.

## Methods

### Parasite culturing

Cam3.II Kelch13 WT and Cam3.II Kelch13 C580Y were generated by genetically editing RF967 (Cam3.II Kelch13 R539T) using zinc-finger nucleases as described in^67^. Cam3.II Kelch13 WT Rpt4 E380*, Cam3.II Kelch13 WT Rpn6 E266K, Cam3.II Kelch13 C580Y β2 C31Y, Cam3.II Kelch13 C580Y β2 C31F, Cam3.II Kelch13 C580Y β5 A20S, and Cam3.II Kelch13 C580Y Rpt5 G319S were obtained from selection studies with the *Plasmodium*-specific proteasome inhibitors WLL and WLW as described in^58^. The abbreviations for these parasites are listed in Table 1. 159–2 parasites were kindly provided by Professor David Fidock (Columbia University Irving Medical Center, New York, NY). 159–2 parasites are Dd2 parasites harboring an episome that contains EGFP under the control of an Hsp70 promoter and a blasticidin resistance cassette. Parasites were cultured as previously described^55^. Briefly, parasites were propagated at 5% hematocrit in O+ red blood cells (RBCs) (Interstate Blood Bank, Memphis, TN) and complete media (RPMI 1640 media supplemented with 0.01 mg/mL gentamicin (Gibco, Billings, MT), 50 mg/mL hypoxanthine (Thermo Fisher Scientific, Waltham, MA), 0.5% Albumax II (Invitrogen, Carlsbad, California)), and 25 mM HEPEs (Fisher Scientific). RBCs were stored at 4°C at 50% hematocrit in an ADSOL solution (2 mM adenine (Alfa Aesar, Haverhill, MA), 111 mM dextrose (Fisher BioReagents, Pittsburgh, PA), 41.2 mM mannitol (Acros Organics, Fair Lawn, NJ), and 154 mM sodium chloride (Fisher BioReagents)^85^. Parasites were grown at 37°C in a Heracell^™^ VIOS 160i CO_2_ Incubator (Thermo Fisher Scientific) at 5% O_2_, 5% CO_2_, and 90% N_2_ (Matheson Gas, Irving, Texas).

### Stage synchronization

For dose response assays, early ring stages (0–3 hpi) were obtained as previously described^55^. Briefly, cultures were exposed to 5% sorbitol (Acros Organics) at 37°C for 10 min and then cultured for 33 h. Then, cultures were incubated with RPMI 1640 supplemented with 14.3 U/mL sodium heparin (Merck, Kenilworth, NJ) at 37°C for 30 min with intermittent vortexing. Cultures were then layered on a 75% Percoll (GE Healthcare, Chicago, IL) density gradient and centrifuging at 4000 rpm (3100 × *g*) for 15 min. The schizont layer (layer immediately above the Percoll) was harvested and washed once with RPMI 1640 supplemented with 14.3 U/mL sodium heparin. Schizonts were cultured in complete media at 2% hematocrit for 3 h. Then, 0–3 hpi rings were obtained following an additional treatment with 5% sorbitol. To obtain a higher protein yield for Western blot experiments, early rings were obtained as described in^86^. Briefly, cultures were treated with 5% sorbitol a total of three times. Cultures were incubated 12 h between first and second treatments, and then 36 h between second and third treatments. Trophozoite stage parasites (26–30 hpi) were obtained using two treatments with 5% sorbitol 12 h apart. Following the second treatment, parasites were cultured for an additional 12 h.

### Drug treatments and lysate preparation

Parasites were synchronized as described above and treated with the indicated compound for the indicated time under hypoxic conditions. DMSO concentration did not exceed 0.2%. Parasites were released from RBCs using 0.15% saponin (Acros Organics) then washed three times with 1 × PBS at 4°C. For Western blots, parasites were lysed with 1% Triton X-100 (Thermo Fisher Scientific), 5% glycerol (Thermo Fisher Scientific), 20 mM MgCl_2_ (Sigma-Aldrich, St. Louis, MO), 200 mM KCl (Sigma-Aldrich), and 25 mM HEPES (pH = 7.4). For proteasome activity assays, parasites were lysed with NP-40 lysis buffer (Thermo Fisher Scientific) supplemented with 500 μM MgCl_2_. Parasite pellets were incubated with the indicated lysis buffer on ice for 15 min with intermittent vortexing. Samples were then centrifuged at 14,000 × *g* for 10 min at 4°C and supernatants were transferred to a new microcentrifuge tube. Protein concentration was determined using a Pierce^™^ BCA assay (Thermo Fisher Scientific). Samples were flash-frozen with a dry ice and ethanol bath and then stored at −20°C.

### Western blots

Parasite lysates were mixed with 4x Laemmli SDS sample buffer (Thermo Fisher Scientific) and boiled at 95°C for 5 min. Equal amounts of parasite proteins, ranging from 2 to 5 μg per lane per blot, were loaded on 4–20% Criterion TGX Stain-Free gels (Bio-Rad, Hercules, CA) and run at 100 V for 1.5 h. Blots were wet transferred at 10 mA at 4°C overnight to PVDF membranes (Immobilon-P, Millipore Sigma, Burlington, MA). Blots were blocked in 3% BSA (Thermo Fisher Scientific) in 1 × TBS-T and then probed with 1:1000 dilutions of primary antibodies overnight at 4°C. Blots were washed 3 times with 1 × TBS-T and then incubated with secondary HRP-conjugated antibodies at 1:10,000 dilutions for 1 h at room temperature. Primary antibodies were used in the following order: phospho-eIF2α (catalog number: 119A11), eIF2α (catalog number: D7D3), K48-linked ubiquitin (catalog number: D9D5), and BiP (catalog number: MRA-1247). The following reagent was obtained through BEI Resources, NIAID, NIH: Polyclonal Anti-Plasmodium falciparum PfGRP78 (BiP), Anti-SGDEDVDSDEL Peptide (antiserum, Rat), MRA-1247. All other primary antibodies were obtained from Cell Signaling Technologies (Danvers, MA). All secondary antibodies were obtained from Invitrogen (Waltham, MA). After washing 4 times with 1x TBS-T, blots were visualized using Immobilon Western Chemiluminescent HRP substate (Millipore Sigma). Blots were stripped with Restore PLUS Western Blot Stripping Buffer (Thermo Fisher Scientific) between antibodies of the same species. Densitometry was performed with ImageJ version 1.53K. Statistical significance was analyzed with GraphPad version 9 using a two-tailed paired *t*-test.

### Proteasome activity assays

OptiPlate-96 black plates (PerkinElmer, Waltham, MA) were placed on ice and 100 μL assay buffer (50 mM Tris pH 7.5, 40 mM KCl, 5 mM MgCl_2_, 0.5 mM ATP (TCI America, Portland, OR), 1 mM DTT (Thermo Fisher Scientific), and 0.5% BSA) was added per well. Then, 10 μg or 20 μg of parasite lysates was added for caspase-like and chymotrypsin-like or trypsin-like assays, respectively. 3 μM Ac-Nle-Pro-Nle-Asp-AMC (Ac-nLPnLD-AMC), 750 nM Ac-Arg-Leu-Arg-AMC (Ac-RLR-AMC), or 6 μM Succinyl-Leu-Leu-Val-Tyr-AMC (Suc-LLVY-AMC) were used to assess caspase-like, trypsin-like, and chymotrypsin-like activity, respectively. All fluorogenic substrates were purchased from Cayman Chemical (Ann Arbor, MI). 1 × PBS was added to obtain a final volume of 300 μL. Samples were mixed and then read on a TECAN Spark (Morrisville, NC) microplate reader pre-warmed to 37°C at 360/480 excitation/emission (ex/em). Readings were taken every 3 min for 2 h or until fluorescence exceeded the detection maxima. To determine activity, relative fluorescence was plotted over time and the slope of the line was determined in Microsoft Excel. At least 3 biological replicates were performed for each substrate. Student *t*-tests were used to determine differences in relative activity.

### Growth inhibition and survival assays

Two-fold serial drug dilutions were performed in 96-well plates (Thermo Fisher Scientific) and parasites were seeded at 1% hematocrit and 0.2% parasitemia in 200 μL total per well. For early ring (0–3 hpi) and trophozoite (26–30 hpi) dose response assays, parasites were treated for 3 h in U-bottom plates, and then drug was washed out. Three to four washes were performed by centrifuging 96-well plates at 1500 rpm for 1 min, removing media, and adding 190 μL media per well. Then, culture was transferred to a new flat-bottom 96-well plate, and plates were incubated for approximately 66 h under normal culturing conditions. For asynchronous dose response assays, parasites were treated for 72 h in flat-bottom plates. Viable parasites were quantified either by flow cytometry or high content imaging^87^. IC_50_ values were calculated in GraphPad Prism version 9.4.1 using non-linear regression analysis. Percent survival for RSAs was calculated by dividing the parasitemia of parasites treated with 700 nM DHA by the parasitemia of mock-treated parasites. For 19S IC_50_ values, outliers were identified and excluded based on a Grub’s test with an alpha = 0.2. At least four independent biological replicates were performed for each assay and statistical significance was examined by Mann-Whitney *U* tests.

### Competition assays

Prior to starting competition assays, 159–2 parasites were grown in media containing 2 μg/mL blasticidin for a minimum of 2 weeks to ensure that > 90% of parasites were EGFP positive. Blasticidin selection pressure was removed prior to and through the duration of competition assays. Parasites of interest were adjusted to 1% parasitemia, and mixed 3:1 with the 159–2 parasite strain. A 1:1 ratio was not used since an initial experiment revealed that 159–2 parasites outcompete Cam3.II parasites within one week. Parasites were cultured in drug-free media at 5% hematocrit and maintained between 0.2 and 7% parasitemia. As a control, wells containing Cam3.II Kelch13 C580Y alone or 159–2 alone were grown concurrently to control for background EGFP fluorescence or loss of EGFP expression in the absence of blasticidin, respectively. No loss in EGFP expression was noted in 159–2 parasites grown in the absence of blasticidin through the duration of competition assays.

Every 2–3 days, the ratio of EGFP-negative to EGFP-positive parasites was assessed by high content imaging. 50 μL of stain containing 2 μg/mL Hoechst 33342 in 1 × PBS was added per well to CellCarrier-96 Ultra Microplates coated with 0.1 mg/mL poly-L-lysine. Then, 0.3 μL of parasite culture was added per well. Cells were stained for 10 min at 37°C and then imaged on an Operetta CLS High-Content Analysis System using the following settings: 20x air objective, non-confocal, binning 2. Channels were captured in the following order: Hoechst (excitation/emission (ex/em) 355–385/430–500), EGFP (ex/em 460–490/500–550), and brightfield. For each channel, focus height was determined, and exposure time and percent power were adjusted so that the intensity of ring stages was approximately 2000–3000 counts/pixels. At least 500 infected RBCs cells were imaged per well. Two technical replicates were performed for each experiment. Images were analyzed using Harmony^®^ 4.9 software with PhenoLOGIC to quantify the percent of EGFP-positive and EGFP-negative parasites. Cultures were maintained until the percent of EGFP-positive parasites in mixed cultures was equal to that of the 159–2 only culture. One generation (*t*) was considered to be equivalent to 48 h. The frequency of EGPF-positive and EGFP-negative are represented as *p*_*t*_ and *q*_*t*_, respectively. As per the equation, $\text{ln}\left(\frac{p_{t}}{q_{t}}\right)=\text{ln}\left(\frac{p_{0}}{q_{0}}\right)+tln\left(\omega \right)$, relative fitness (ω) was calculated by determining the slope (*m*) of $\text{ln}\left(\frac{p_{t}}{q_{t}}\right)$ versus *t* and solving for ω = e^*m* 88^. The selection coefficient (*s*) was determined using the formula *s* = ω − 1. Statistical significance was performed using a Student’s *t*-test. Two biological replicates were perfomed.

### Drug compounds

DHA was purchased from Sigma Aldrich (St. Louis, MO). OZ439 was kindly provided by Professor Jonathan Vennerstrom (University of Nebraska Medical Center). Methylene blue and chloroquine were purchased from Thermo Fisher Scientific. Epoxomicin was purchased from APExBIO (Houston, TX). b-AP15 was purchased from Calbiochem (San Diego, CA). WLL was kindly provided by Professor Mathew Bogyo (Stanford School of Medicine). Blasticidin was purchased from MP Biomedicals (Santa Ana, CA).

## Figures and Tables

**Table 1:** Parasite susceptibilities to proteasome inhibitors and DHA.

	Drug Sensitivity				RSA %survival (mean ± S.E.M.)
Parasite Name	Abbreviation	WLL	WLW	DHA	
Cam3.II K13 WT	WT	S^a^	S^a^	S^b,c^	0.3 ± 0.3^b^
Cam3.II K13 R539T	R539T	S^a^	S^a^	R^b,c^	34.1 ± 4.1^b^
Cam3.II K13 C580Y	C580Y	S^a^	S^a^	R^b,c^	11.4 ± 1.7^b^
Cam3.II K13 C580Y β2 C31Y	β2 C31Y	HS^a,b^	R^a,b^	S by IC50; R by RSA^b^	6.1 ± 1.3^b^
Cam3.II K13 C580Y β2 C31F	β2 C31F	HS^a,b^	R^a,b^	S by IC50; R by RSA^b^	11.5 ± 1.1^b^
Cam3.II K13 C580Y β5 A20S	β5 A20S	R^a,b^	HS^a,b^	R^b^	15.7 ± 0.8^b^
Cam3.II K13 WT Rpt4 E380*	Rpt4	S^a^	R^a^	S^d^	
Cam3.II K13 WT Rpn6 E266K	Rpn6	S^a^	R^a^	S^d^	
Cam3.II K13 C580Y Rpt5 G319S	Rpt5	S^a^	R^a^	S by IC50; R by RSA^d^	8.5 ± 0.83^d^

HS: hypersensitive, S: sensitive, R: resistant. References documenting susceptibilities are provided.

a Ref 57 Stokes, B. H. et al. Covalent *Plasmodium falciparum*-selective proteasome inhibitors exhibit a low propensity for generating resistance in vitro and synergize with multiple antimalarial agents. PLoS Pathog 15, e1007722, doi:10.1371/journal.ppat.1007722 (2019).

b Ref 55 Rosenthal, M. R. & Ng, C. L. A proteasome mutation sensitizes *P. falciparum* Cam3.II K13(C580Y) parasites to DHA and OZ439. *ACS Infect Dis*, doi:10.1021/acsinfecdis.0c00900 (2021).

c Ref 67 Straimer, J. et al. K13-propeller mutations confer artemisinin resistance in Plasmodium falciparum clinical isolates. Science 347, 428–431, doi:10.1126/science.1260867 (2015).

d Values reported in this manuscript.

## Inclusion and Diversity

One or more of the authors of this paper self-identifies as an underrepresented ethnic minority in their field of research or within their geographical location. One or more of the authors of this paper self-identifies as a gender minority in their field of research. One or more of the authors of this paper received support from a program designed to increase minority representation in their field of research.
